# A Phase I Study of sequences of the CDK4/6 Inhibitor, Ribociclib Combined with Gemcitabine in Patients with Advanced Solid Tumors

**DOI:** 10.21203/rs.3.rs-4261257/v1

**Published:** 2024-04-25

**Authors:** Mahesh Seetharam, Aurora Norman, Jacob Allred, Jianping Kong, Mateusz Opyrchal, Wen Wee Ma, Yanyan Lou, Grace K Dy, Amit Mahipal, John Weroha, Andrea Wahner-Hendrickson, Joel M. Reid, Alex A. Adjei

**Affiliations:** Mayo Clinic; Mayo Clinic; Mayo Clinic; Mayo Clinic; Indiana University; Cleveland Clinic; Mayo Clinic; Roswell Park Comprehensive Cancer Center; University Hospitals at Case Western University; Mayo Clinic; Mayo Clinic; Mayo Clinic; Cleveland Clinic

## Abstract

**Background:**

Based on preclinical data showing addition of CDK4/6 inhibitors to gemcitabine is synergistic, ribociclib was evaluated in combination with gemcitabine to determine the maximum tolerated dose (MTD) and dose limiting toxicities (DLT).

**Methods:**

In this single arm multicohort phase I trial, we evaluated the safety and efficacy of Ribociclib plus Gemcitabine in patients with advanced solid tumors. Patients received Gemcitabine intravenously on days 1 and 8 followed by Ribociclib days 8–14, with treatment repeated every 3 weeks.

**Results:**

The study enrolled 43 patients between October 2017 and September 2019. The escalation phase (19 patients) determined the MTD and recommended phase II dose (RP2D) to be ribociclib 800mg daily and gemcitabine 1000mg/m2 for the expansion phase (24 patients). One patient experienced Grade 4 thrombocytopenia. Eleven patients experienced Grade 3 adverse events (AE), the most common being neutropenia, thrombocytopenia, and anemia. No partial or complete responses were observed. 15/22 (68%) of efficacy evaluable patients who received the MTD achieved best response of stable disease.

**Conclusions:**

The addition of Ribociclib to Gemcitabine was tolerated well and yielded stability of tumors in both cohorts. Ribociclib and gemcitabine could have synergistic activity in certain tumor types, and our data provides support for the combination.

**Clinical Trial Registration:**

NCT03237390

## Introduction

Ribociclib is a kinase inhibitor which inhibits CDK4/Cyclin D1 and CDK6/Cyclin, thereby inducing G1-phase cell cycle arrest and allowing for therapeutic targeting of cancer(1–6). CDK4/6 inhibitors have been approved for treatment of hormone receptor positive metastatic breast cancer in combination with hormonal blockade agents (7–10). While ribociclib has limited single agent activity(4),combining CDK4/6 inhibitors with chemotherapy has shown promise in lung and breast cancers(11, 12).

Combining CDK4/6 inhibitor with conventional cell cycle-specific chemotherapy may potentiate the antitumor effect of each therapeutic agent and delay resistance development due to the non-overlapping mechanism of actions of these agents. Single agent gemcitabine is the standard treatment for many solid tumor malignancies including NSCLC, breast cancer and pancreatic cancer. Gemcitabine is a nucleoside analogue that exhibits cell phase specific antitumor activity, primarily killing cells undergoing DNA synthesis (S-phase), and also blocking the progression of cells through the G1/S-phase boundary. We anticipated that combination of ribociclib and gemcitabine may enhance the anti-tumor activity of each agent.

Based on the mechanisms of action of ribociclib and gemcitabine within the cell cycle, we originally hypothesized the optimal sequencing of this combination to be administration of ribociclib followed by gemcitabine. A small Phase 1 trial opened at Roswell Park Comprehensive Cancer Center (NCT02414724) consisted of treatment with ribociclib once daily on Days 1–14 and gemcitabine IV on Day 1 and 8 of a 21 day treatment cycle. However, this study was discontinued early when significant toxicity, predominantly myelosuppression, made concomitant administration of efficacious doses of ribociclib and gemcitabine in this order infeasible.

Additional preclinical work showed that in H1299 lung cancer cell line, gemcitabine treatment followed by either ribociclib alone or a subsequent dose of gemcitabine combined with ribociclib significantly enhanced cytotoxic effects (Supplemental Fig. 1), while preceding ribociclib treatment abrogated gemcitabine’s cytotoxicity. This data contradicted the original hypothesis regarding optimal sequencing of ribociclib and gemcitabine and indicated that gemcitabine

Supplemental Fig. 1. Annexin V/7-ADD apoptosis analysis in H1299 cells after treatment with single agent or different sequential combinations. Each value is a mean ± SD (n = 3–4). * indicates P < 0.0001, where the difference is statistically significant in comparison with DMSO control, as determined by one-way ANOVA followed by Dunnett’s test. Methods used to produce this assay have been published previously.

should precede ribociclib within a treatment cycle for improved efficacy and to also potentially mitigate the issue of profound myelosuppression seen in the phase 1 clinical trial at Roswell Park.

We describe a phase I clinical trial at Mayo Clinic combining gemcitabine and ribociclib in patients with progressive metastatic carcinomas which changed the administration sequence of these drugs within a treatment cycle for safety and efficacy based on the preclinical and early clinical data described above.

## Methods

### Clinical study design

This single-center, nonrandomized, dose escalation, open-label, investigator-initiated phase I study enrolled patients at Mayo Clinic between patients between October 2017 and September 2019. Eligible patients were those with advanced or metastatic solid malignancy for which no standard treatment option existed that would confer clinical benefit and who had life expectancy of 12 weeks or greater at the time of evaluation;≥18 years of age with biopsy-confirmed malignancy; ECOG PS of 0 or 1; adequate bone marrow and organ function; QTcF of <450msec; and measurable disease per RECIST. All their side effects from previous treatments or surgery must have resolved to grade 1 or better according to CTCAEv4.03. A negative pregnancy test within 7 days prior to study drug was required in women of childbearing potential; contraception practices were required for women (or men sexually active with women) who were of child-bearing potential. All patients had to be able to swallow the ribociclib capsule.

The study excluded patients who had received anti-cancer chemotherapy, immunotherapy, radiotherapy, or investigational agents within 28 days prior to registration or limited field radiation for palliation or major surgery ≤14 days prior to registration or if ≥25% bone marrow was irradiated. Also excluded were patients who had impaired GI function or disease that may affect absorption of study drugs; baseline neuropathy of > grade 2; active, clinically serious infection or known history of HIV infection; other serious uncontrolled medical conditions; or hypersensitivity to any of the excipients of ribociclib. If they had known central nervous system (CNS) involvement they were excluded unless they were at least 4 weeks from prior therapy completion (clinically stable CNS disease without steroids and/or enzyme-inducing anti-epileptic medications for brain metastasis were allowed).

Prohibited concomitant medications included strong inducers or inhibitors or CYP3A4/5, drugs with narrow therapeutic window and predominantly metabolized through CYP3A4, medications known to cause QT prolongation or induce Torsades de Pointes and herbal preparations.

Two study phases were planned: a dose escalation phase and an expansion phase. The dose escalation phase was based upon the standard modified Fibonacci 3+3 study design and projected to accrue up to 30 patients. The four escalation dosing levels (Supplemental Table 1) were prespecified based on the anticipation that the main DLT would be myelosuppression due to it being a common AE of both study agents. The starting dose level used reduced standard clinical doses of both study agents. Each subsequent level escalated the dose of one of the study agents. The rationale used to determine the anticipated acceptable maximum combined dosing for the final escalation dose level (DL4) was based on a standard clinical dosing of gemcitabine (1000 mg/m2) and 800 mg/day of ribociclib (one dose level greater than standard clinical dosing of 600 mg/day). The dose level at which MTD was determined would be identified as the RP2D and used for the expansion phase. The expansion phase planned to enroll an additional 20 patients to reasonably evaluate the PK and other correlative science endpoints as well as DLTs to elaborate the decision regarding the appropriateness of the choice for the MTD from the dose escalation phase. Investigators and patients were not blinded to study treatment.

All participants granted written informed consent according to federal and institutional guidelines. The trial was conducted with approval of Mayo Institutional Review Board. The study is registered with ClinicalTrials.gov with the identifier NCT03237390.

### Study treatment

Treatment cycles were 21 days in length. Gemcitabine was administered intravenously on cycle days 1 and 8 and ribociclib taken orally once daily on days 8–15, followed by a 7-day rest. In order to minimize the inter-patient variability of PK assessments, participants took their first cycle dose of ribociclib immediately before gemcitabine infusion on day 8. Novartis provided ribociclib. The initial cohort in the dose escalation phase started at Dose Level 1 and subsequent patient cohorts were enrolled based on the safety and tolerability of the preceding dose level cohort as outlined in Supplemental Table 1.

Adverse events (AE) were graded according to the National Cancer Institute Common Toxicity Criteria version 4 (NCI CTCAE v4.0). When patients experienced Grade 3 or Grade 4 treatment related toxicity or intolerable Grade 2 toxicity despite optimal supportive care, treatment might be delayed and/or dose reduced. In the event of multiple toxicities, dose modification was based on the worst toxicity observed. For any AE ≥ Grade 2, ribociclib was dose interrupted until recovery to Grade ≤ 1, except for Grade 2 anemia or neutropenia for which no dose adjustment was required. When a patient required dose modification of ribociclib due to protocol-guided AE, the dose was reduced by 200 mg per day. Ribociclib dose reductions to the next lower dose level were required if a patient experienced a recurrence of Grade 3 thrombocytopenia, a Grade 3 neutropenia that took > 7 days to resolve, first Grade 4 neutropenia, first Grade 3 febrile neutropenia, or any Grade 3 nonhematological AE. Ribociclib was discontinued if a patient developed a Grade 4 anemia, febrile neutropenia, or non-hematological toxicity. Gemcitabine was postponed on Day 1 without dose adjustment on resumption for thrombocytopenia with platelet count < 100 × 10^9^/L until platelets recovered to above that threshold; neutropenia Grade 2/3 until recovery to ≤ Grade 1; anemia Grade 3 until recovery to Grade ≤ 2; and non-hematological AE Grade 2 until recovery to Grade ≤ 1. Gemcitabine was dose reduced after recovery from Grade 4 thrombocytopenia and neutropenia or Grade 3 febrile neutropenia. Gemcitabine was discontinued if a patient developed Grade 4 febrile neutropenia or anemia.

### Endpoints

The primary objectives were to describe the dose-limiting toxicities (DLT) and identify the MTD and recommended Phase II dose (RP2D) of the combination of ribociclib and gemcitabine in patients with advanced solid tumors. The MTD was defined as the maximum dose level at which ≤ 1/6 patients had DLT in the dose escalation phase. All patients who received the combination of study drugs within the first cycle were considered evaluable for toxicity.

DLT for this trial were defined as grade 4 neutropenia > 7 consecutive days; grade 4 thrombocytopenia or grade 3 with bleeding; grade 3 or 4 febrile neutropenia; QTc interval ≥ 501ms on ≥ 2 separate EKGs; cardiotoxicity or troponin ≥grade 3 or clinical signs of cardiac disease such as unstable angina or myocardial infarction; vomiting ≥ grade 3 over 48 hours despite optimal anti-emetic therapy; diarrhea ≥ grade 3 over 48 hours despite optimal anti-diarrheal therapy; bilirubin ≥ grade 2 for over 7 consecutive days or grade 3; ALT ≥grade 2 with a ≥grade 2 bilirubin elevation of any duration in absence of liver metastases, ALT≥ grade 3 for more than 4 consecutive days; grade 4 ALT or AST; grade 4 serum alkaline phosphatase >7 consecutive days; serum creatinine ≥grade 3; any non-hematologic events ≥grade 3 (excluding alopecia; grade 3 fatigue <5 days, grade 3 fever or infection without neutropenia < 5 days duration; grade 3 laboratory abnormalities responsive to oral supplementation or deemed by the investigator to be clinically insignificant). Persistent, intolerable treatment related toxicities which delayed treatment for >14 days, and failure to receive at least 80% of the scheduled doses due to treatment related toxicity (except when treatment delay is due to sub-optimally managed nausea, vomiting or diarrhea) were also considered DLT. If a patient did not have a DLT but could not complete at least 80% of ribociclib and 2 doses of gemcitabine, then they would be replaced. Safety evaluations were completed throughout the study and a follow-up safety evaluation included AE assessment and review of concomitant medications and occurred 30 days (+/− 3 days) after the last dose of study drug or until resolution of any drug related toxicities.

The secondary objectives were to describe pharmacokinetics, antitumor activity of the combination of ribociclib and gemcitabine, and correlative biomarker analysis. Secondary endpoints of efficacy included response rate (RR), progression free survival (PFS), and short-term survival. Tumors were measured at baseline and prior to or on Cycle 3 Day 1, then prior to or on Day 1 of every other cycle thereafter. RECIST 1.1 was used to assess and document RR in terms of complete response (CR), partial response (PR), stable disease (SD), and progressive disease (PD). PFS was defined as time from enrollment to disease progression determined either clinically or radiographically. Three months after patients terminated treatment, follow-up short-term survival information was collected without further follow-up afterwards.

### Pharmacokinetics

Serial whole blood samples for pharmacokinetic analysis of ribociclib were collected in two 2 mL EDTA tubes during the first cycle of treatment, 30 minutes before and 0.5, 1, 2, 4, 6, and 8 hours after administration of ribociclib on day 8 and day 14 of the first cycle of treatment. Plasma was isolated by centrifugation at 1,500 × g for 10 minutes within 30 minutes following blood collection and aliquots (2 ml each) were placed in ice immediately and then stored frozen at −70°C or below until analyzed. The plasma concentration of ribociclib was determined using a liquid chromatography/tandem mass spectrometry (LC-MS/MS) assay. Quality assurance was maintained by injecting quality control samples each time patient samples were assayed.

Plasma concentration-time data were analyzed by standard non-compartmental methods using the program WinNonlin (Pharsight, Mountain View, CA). The areas under the plasma concentration time curve (AUC) on day 8 and day 14 were calculated by trapezoidal approximation. The accumulation ratio (R) was calculated as the ratio of day 14 AUC_0–8h_ versus the day 8 AUC_0–8h_. The apparent elimination half-life (t_1/2_) was calculated as −(0.693*τ)/ln((R−1)/R) where R is accumulation ratio and τ is the dosing interval (24 hours). Since a 24-hour blood sample was not drawn on day 14, the 24-hour plasma concentration after ribociclib administration was estimated to be equivalent to the pre-dose concentration based on the assumption that steady-state was reached on day 14, and the AUC over the 24-hour dosing interval on day 14 (AUC_t_) was calculated by trapezoidal approximation. Oral steady-state clearance (CL_SS_/F) was calculated using the equation, CL_SS_/F = Dose/AUC_t_, where dose is the administered dose of ribociclib. Standard descriptive statistics were used to summarize plasma ribociclib PK parameters.

### Biomarker correlates

Optional tissue samples (archival) when available or biopsy if feasible were collected to evaluate correlation between CDK2/4/6, Cyclin D1 and Cyclin D3amplification, RB and P16 expression in archived and/or biopsied tumor tissue and treatment response. Tissue samples were labeled as “primary”, “metastatic” or “recurrent” upon submission. The tissue could be archived as either a paraffin-embedded tumor block or unstained slides (a minimum of 10 slides, 5 μ thickness with tumor content of 20% or higher as assessed by hematoxylin and eosin).

### Statistical analysis

No power analysis was performed as the study was not powered for any endpoints. These data were analyzed descriptively (simple counts, percentages, and median) to assess the primary and secondary endpoints.

## Results

### Study population

**Figure 1:**
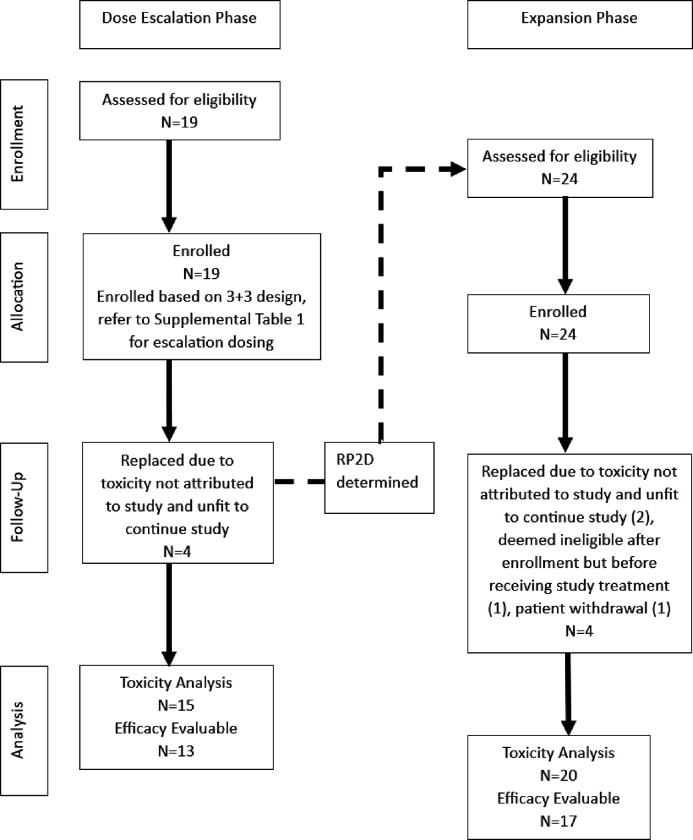
Consort diagram. Enrollment, Allocation, Follow-Up, Analysis

A total of 43 patients were enrolled on the study between October 2017 to October 2019 (Table 1). The first 19 patients were enrolled in the dose escalation phase between October 2017 and December 2018 and 24 patients were enrolled in the expansion phase (MTD) of the study between February 2019 and October 2019 (Figure 1). Median age of both cohorts was similar at 62 and 60 years for escalation and expansion, respectively. The majority of patients in both cohorts had either lung or pancreatic cancer, as outlined in Table 1. The proportion of patients who had received and progressed on gemcitabine prior to enrollment in our study was 37% in the escalation cohort and 50% in the expansion cohort.

### Safety

During the dose escalation phase, no patients experienced treatment-related DLT at any of the 4 dosing levels. Once study enrollment reached the highest dose level, 6 total patients were enrolled to meet criteria for MTD definition prior to proceeding to expansion phase enrollment (Table 2).

In the escalation phase, 4/19 patients developed AE prior to receiving ribociclib in cycle 1 that rendered their performance status unfit to continue the study, and thus they were considered inevaluable for the purpose of defining the MTD and replaced (Figure 1). In the expansion phase, 4/24 patients were replaced due to being deemed ineligible after enrollment but prior to receiving any study drug (1), patient withdrawal from study prior to receiving study drug combination (1), or developing AE prior to receiving ribociclib in cycle 1 that rendered them unfit to continue on study (2). Of the remaining 20 evaluable patients, 3 were noted to have DLT, however only one of those was treatment-related (thrombocytopenia). The other 2 patients had treatment-unrelated DLT (hyperbilirubinemia, dyspnea) combined with prior study-treatment delays that made them ineligible to continue on study. The patient who died while on study had not received study treatment for 2 cycles prior due to declining performance status and final cause of death was sudden cardiac arrest unlikely to be related to study treatment. The safety results from the expansion cohort did not alter the choice of MTD/RP2D determined by the escalation phase.

A total of 35 patients were included in toxicity assessment after the 8 patients who were replaced were excluded. As mentioned before, 6 of those excluded patients developed AE that were not attributable to the combination of study drugs, but rather gemcitabine alone, as they did not receive any ribociclib in cycle 1 (Figure 1). Toxicities that were considered at least possibly related to either ribociclib or gemcitabine are shown in Table 3.

To precisely represent AE rates according to dose level, we grouped the DL4 patients with the expansion cohort patients (n=26) as they all received the same MTD. All Grade 3/4 AE occurred in the DL4+Expasion cohort patients. Eleven patients experienced Grade 3 AE, all of which were hematological. The most common Grade 3 AE were neutropenia (7), thrombocytopenia (3), and anemia (3). Some patients experienced more than one Grade 3 AE. One patient experienced Grade 4 thrombocytopenia. The most frequent AE were fatigue and nausea.

### Treatment response

Out of the 35 patients who were enrolled and not replaced, 30 were evaluable for efficacy: 3 in DL1, 2 in DL2, 3 in DL3, and 22 in DL4+expansion. The 5 patients not evaluated for disease response came off study for reasons other than disease progression prior to first tumor reassessment imaging to be done prior to Cycle 3 according to protocol. These reasons included patient withdrawal from study (4) and study treatment DLT (1).

**Figure 2:**
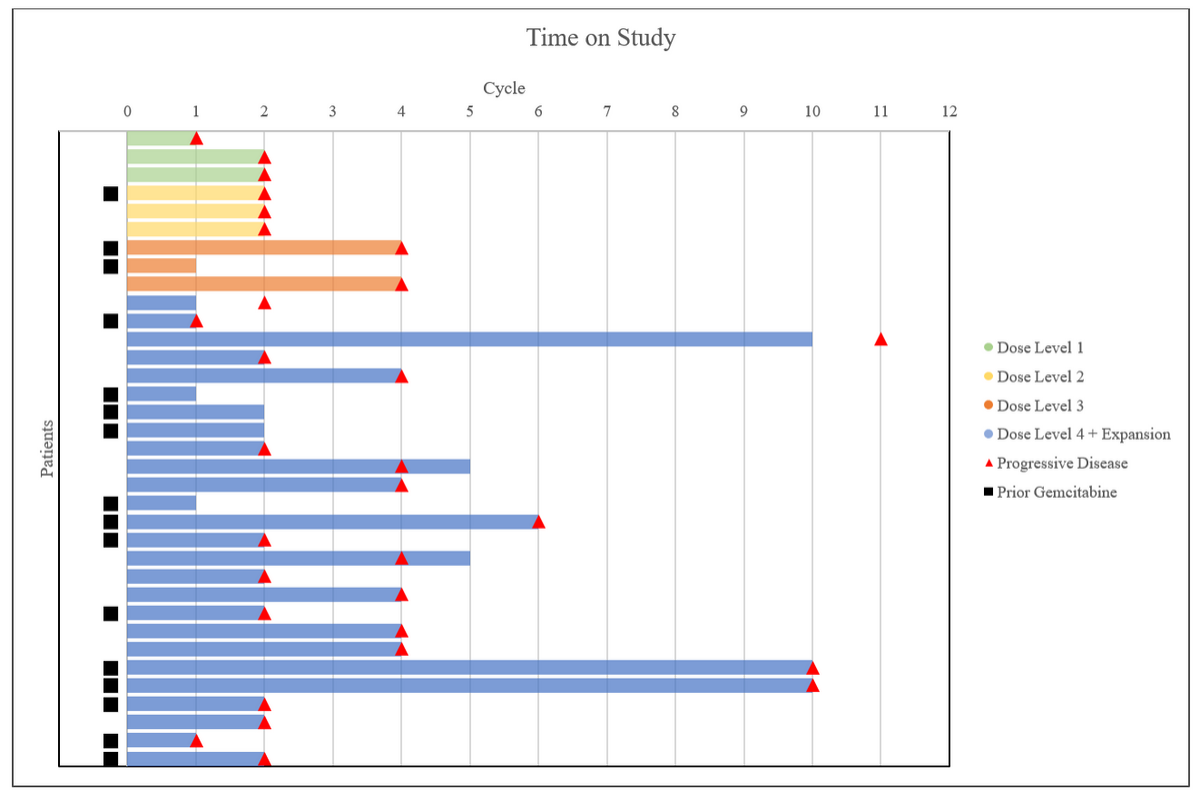
Time on study, in terms of treatment cycles for each patient (n=35). Dose level cohorts are shown as DL1(green), DL2(yellow), DL3(orange, and DL4+Expansion(blue). Cycle at which disease progression was determined either clinically or radiographically is marked by a red triangle. Patients who had previously been exposed to gemcitabine treatment and progressed prior to participation in this study are marked by a black square.

No partial or complete responses were observed. Stable disease (SD) on evaluation began to be achieved at DL3. Best response of SD occurred in 2/2 (100%) patients in DL3 and 15/22 (68%) patients in DL4+Expansion (Table 4, Figure 2). Median PFS of DL4+Expansion group was 2.45 months.

The median number of cycles received by the efficacy evaluable DL4+Expansion group was 3. The 3 longest patients on study, who each received 10 cycles of MTD therapy, had tumor types of uterine carcinosarcoma, lung, and hepatobiliary. Two of them had progressed on gemcitabine prior to study enrollment (lung and hepatobiliary). Otherwise, there was no obvious pattern between prior gemcitabine therapy and disease progression (Figure 2). In the DL4+expansion group, of the 15/22 who had best response of SD, 5 (33%) had received prior gemcitabine. All efficacy-evaluable patients did eventually demonstrate disease progression, necessitating study treatment end.

### Pharmacokinetics

Ribociclib pharmacokinetics were characterized in 34 patients who received ribociclib in combination with gemcitabine. The dose escalation phase enrolled 11 patients (n=3 for each of dose escalation level DL1 to DL3; n=6 for DL4), and 15 patients received expansion dosage. Pharmacokinetic parameters of LEE011 by dose level are provided in Supplemental Table 2. The peak plasma concentration was achieved 4 hours (Range, 0.5 – 6.1 hours) after drug administration. Substantial variability was observed for peak plasma concentration (C_max_) and drug exposure (AUC_0–8h_) as shown in Supplemental Figure 2, however, the increase in mean values was proportional to dose (Supplemental Table 2). Following consecutive oral doses on Days 8–14, 2.1-fold accumulation of ribociclib was observed leading to an estimated half-life of 25.9 hours (range, <6 – 71.7 hours).

### Follow-up

Follow-up information, showed that 21 (70%) of all efficacy-evaluable study patients were still alive at short-term survival assessment occurring either 3 months after study treatment cessation or last follow-up while on treatment if they were not able to be contacted to assess survival follow-up. (Table 4). In the DL4+Expansion group, 16 patients (73%) were alive at short-term survival assessment, 2 of these patients had survival assessment based on most recent assessment while on treatment due to being out of contact at the 3-month post-treatment cessation timepoint.

## Discussion

In this phase I study we show that ribociclib can be safely administered with gemcitabine. We determined the MTD to be 800mg daily for 7 days when co-administered with gemcitabine at standard dosing of 1000mg/m2 every 21 days. The DLT were primarily Grade 3 hematologic, but manageable with appropriate supportive care and dose adjustment as outlined in study protocol. The main reason patients came off study was due to disease progression rather than intolerable treatment AE.

There are multiple potential mechanisms by which chemotherapy may impact the activity of CDK2 and CDK4/6 complexes that might be expected to enhance the efficacy of CDK4/6 inhibitor. Chemotherapy may induce rapid degradation of CDC25A or rapid induction of CDK-2 inhibitor P21CIP1. Conversely, if the chemotherapy induces S-phase block, that might result in degradation of cyclin D1 and inhibition of CDK4/6 complexes(13).

Use of concurrent chemotherapy with CDK4/6 inhibitors may have differential outcomes based on the type of chemotherapy and its mechanism of action. Chemotherapy impacts CDK biology in different ways which could enhance the response to CDK4/6 inhibition(14). Based on the canonical action of chemotherapy and inhibitors of CDK4/6, it is rationale to sequence treatment with chemotherapy and CDK4/6 inhibitors. Chemotherapy results in cytotoxicity and killing of tumor cells and targeted therapy with CDK4/6 antagonists prevent expansion of cells which escape cytotoxicity. In triple negative breast cancer, pretreatment of triple negative breast cancer cells treated with CDK4/6 inhibitors followed by chemotherapy improved response to paclitaxel(15). In another phase I study combining paclitaxel and palbociclib alternating sequential palbociclib and paclitaxel in patients with Rb + advanced breast cancer was safe and feasible without additional toxicity with clinical benefit rate of 55% at the recommended phase 2 dose level(16). Recent literature also indicates cessation of chemotherapy drugs that target the DNA replication results in DNA-damaged cells entering cell cycle, which primes them to be more sensitive to CDK4/6 inhibition(17).

Our pharmacokinetic studies enhanced the understanding of ribociclib in this novel combination regimen. Ribociclib T_max_ was similar to that reported in previous other adult and pediatric phase I studies(4, 18, 19). The maximum concentration and drug exposure high inter-patient variability on day 8, however, the variation became less on Day 14 once the drug reached the steady state. C_max_ and AUC_0 – 8h_ generally increased with the increasing dose of ribociclib. Median accumulation of ribociclib after 7 days administration was 2.1-fold, which is consistent with other findings(4, 18). It indicated that comparison of ribociclib treatment alone, the plasma maximum concentration and exposure of ribociclib were not affected by the co-administration with gemcitabine.

While the trial was not designed to evaluate clinical response, we saw stabilization of disease in the efficacy evaluable DL4 + Expansion group for a median of 3 cycles in this heavily pre-treated heterogenous patient population. Due to low numbers of patients, interpretation of median PFS change in relation to dose level increase is not robust, but there appears to be a hint of increased linear trend of improving median PFS correlating to increases in ribociclib and gemcitabine combination dose levels. Given the heterogeneity of tumor types in our study, the median PFS we observed of 2.45 months is difficult to interpret in relation to usual expected PFS of patients with a certain tumor type refractory to multiple lines of therapy.

The MTD dose of ribociclib was higher per day but of shorter duration in a cycle than the dose approved to treat metastatic hormone receptor positive breast cancer in combination with anti-hormone therapy, which represents the modification in our protocol after the preliminary data shown by the small feasibility study at Roswell Park Cancer Center was halted after the manifestation of significant overlapping toxicities of bone marrow suppression with gemcitabine. It is possible that an alternative treatment schedule could be explored in the future that would show clinical response. Notably, the revised protocol cut the treatment duration of ribociclib in half per cycle, so patients received 7 days of ribociclib per cycle rather than 14. A possible modification for consideration in future studies that could balance the overlapping myelosuppressive toxicities of both ribociclib and gemcitabine while providing adequate systemic drug exposure per treatment cycle would be to extend the treatment cycle to 28 days, administer gemcitabine on days 1 and 8, and extend ribociclib treatment to days 8–21 followed by one week for recovery prior to the next cycle. With this design, the expected neutropenic nadir from gemcitabine would occur at Day 15 with recovery around Day 21, and the median time to ribociclib-induced neutropenia is 16 days (which would be Day 24 of a cycle if ribociclib is started Day 8), suggesting patients could recover from gemcitabine-induced neutropenia before the neutropenic effects of ribociclib would be expected to occur.

Additionally, use of predictive biomarkers could be explored in the future to identify what patient population might benefit the most from CDK4/6 inhibitors. While hyperphosphorylated Rb activates cell division and thus provides an excellent target for CDK4/6 inhibitors to block Rb phosphorylation, other tumors demonstrate loss of Rb function and therefore will not respond as well to CDK4/6 inhibition due to deficient target presence(20, 21). Rb status and CDK4/6 expression of tumors should be evaluated in future trials to correlate with treatment response. For example, in a case study published in JCO Precision Oncology, a patient with refractory osteosarcoma had genomic testing showing CDK6 amplification with no inactivating alterations or loss of RB1, leading the oncology team to choose combination ribociclib and gemcitabine treatment based on our phase I clinical trial(22). The time to progression in this patient was 9 months, which was considerably longer than that of most patients treated with gemcitabine (with or without docetaxel) for relapsed/refractory osteosarcoma (median 2–4 months).

There is significant interest in utilization of CDK4/6inhibitors including Ribociclib as evidenced by a vast number of clinical trials of ribociclib based novel combinations. The CDK4/6 inhibitors can not only target the tumor cells directly but also the tumor microenvironment, suggesting future work combining these agents with immunotherapies (23, 24).

In conclusion, the combination of ribociclib with gemcitabine showed synergistic activity in certain tumor types in preclinical cell studies but may have antagonistic activity in others based on specific circumstances such as tumor type, the sequencing pattern of chemotherapy and targeted therapies and Rb status. Prior treatment with gemcitabine was not predictive of response or resistance to treatment with gemcitabine and ribociclib. It is possible that the sequencing of gemcitabine followed by ribociclib was beneficial for some patients and tumor types but not in others. Use of biomarkers such as Rb status, and activity of CDK2 and CDK4/6 complexes may help to select patients who may respond better to the combination of gemcitabine and ribociclib. This approach may serve as a maintenance therapy using CDK4/6 inhibitor to target residual cells that survive chemotherapy cytotoxicity.

## Figures and Tables

**Table 1.** Demographics and baseline characteristics.

Patient characteristics	Dose-escalation cohort (n=19)	Dose-expansion cohort (n=24)
		
Median Age, y (range)	62 (26, 74)	60 (27, 79)
Gender, *n* (%)		
Female	13 (68.4%)	12 (50%)
Male	6 (31.6%)	12 (50%)
Race, *n* (%)		
Asian	0 (0%)	1 (4.2%)
White	19 (100%)	22 (91.7%)
Uknown	0 (0%)	1 (4.2%)
ECOG performance status, *n* (%)		
0	6 (31.6%)	10 (41.7%)
1	14 (68.4%)	14 (58.3%)
Prior Gemcitabine Therapy, *n* (%)		
Yes	7 (36.8%)	12 (50%)
No	12 (63.2%)	12 (50%)
Tumor Type, *n* (%)		
Lung	6 (32%)	5 (21%)
Pancreas	5 (26%)	7 (29%)
Cholangiocarcinoma	1 (5.2%)	0
Hepatobiliary	1 (5.2%)	2 (8%)
Breast	1 (5.2%)	2 (8%)
Ovary	1 (5.2%)	2 (8%)
Uterine Carcinoma	1 (5.2%)	0
Head and Neck	1 (5.2%)	0
Melanoma	1 (5.2%)	0
Sarcoma	1 (5.2%)	0
Thorax	0	1 (4.2%)
Small Bowel	0	1 (4.2%)
Pelvis	0	1 (4.2%)
Cervix	0	1 (4.2%)
Nerve Sheath Tumor	0	1 (4.2%)
Unknown Primary	0	1 (4.2%)

**Table 2.** Accrual summary

Dose level	Accrual period	No. enrolled pts	No. cancelled/replaced pts	No. evaluable pts	DLTs
1	10/6/17–11/22/17	5	2	3	-
2	1/22/18–4/20/18	4	1	3	-
3	6/5/18–8/22/18	4	1	3	-
4	10/4/18–12/21/18	6	0	6	-
MTD (expansion)	2/4/19–10/15/19	24	4	20	3

**Table 3a.** Treatment-related adverse events (AE) categorized as hematological or non-hematological

Patients with at least one:	Dose Level 1–3 (n=9)	Dose Level 4 + Expansion cohort (n=26)
Hematological AE, n (%)		
Grade 3+	0	11 (42)
Grade 4+	0	1 (4)
Non-hematological AE, n (%)		
Grade 3+	0	0
Grade 4+	0	0

**Table 3b.** Specific possible treatment-related AE

	Dose Level 1–3 (n=9)			Dose Level 4 + Expansion cohort (n=26)		
AE, n (%)	Grades 1–2	Grade 3	Grade 4	Grades 1–2	Grade 3	Grade 4
Fatigue	0	0	0	9 (35)	0	0
Nausea	1 (11)	0	0	17 (65)	0	0
Neutropenia	0	0	0	1 (4)	7 (27)	0
Leukopenia	0	0	0	2 (8)	5 (19)	0
Thrombocytopenia	0	0	0	2 (8)	3 (12)	1 (4)
Anemia	1 (11)	0	0	1 (4)	3 (12)	0
Vomiting	0	0	0	6 (23)	0	0
Diarrhea	1 (11)	0	0	3 (12)	0	0
Dyspnea	1 (11)	0	0	2 (8)	0	0
Maculopapular Rash	0	0	0	4 (15)	0	0
Pruritis	0	0	0	2 (8)	0	0
Oral mucositis	0	0	0	1 (4)	0	0
Edema	0	0	0	1 (4)	0	0
Fever	0	0	0	1 (4)	0	0
Flu-like symptoms	0	0	0	1 (4)	0	0
Constipation	0	0	0	1 (4)	0	0

**Table 4.** Summary of best response rates, median PFS, and short-term survival rate in efficacy evaluable patients.

Dose Level Group	Best Response		Median PFS (months)	Short-term Survival Rate, n(%)
	SD, n(%)	PD, n(%)		
1 (n=3)	0	3 (100)	1.43	2 (66)
2 (n=3)	0	3 (100)	1.57	1 (33)
3 (n=2)	2 (100)	0	3.27	2 (100)
4 + Expansion (n=22)	15 (68)	7 (32)	2.45	16 (73)

## Data Availability

Raw data for this study were generated at Mayo Clinic in Rochester, MN. Data supporting the findings of this study are available from the corresponding author upon request.
